# Incorporating natural variation into IVF clinic league tables: The Expected Rank

**DOI:** 10.1186/1471-2288-9-53

**Published:** 2009-07-16

**Authors:** Hester F Lingsma, Marinus JC Eijkemans, Ewout W Steyerberg

**Affiliations:** 1 Department of Public Health, Erasmus MC, Rotterdam, the Netherlands

## Abstract

**Background:**

Rankings based on outcome are often used to present health care provider performance. These rankings do however not reflect that part of the variation in outcome between providers is caused by natural variation, and not by any differences in quality of care. The aim of this study is to compare standard methods for ranking with a novel method that takes into account natural variation.

**Methods:**

We used data on the number of treatment cycles and the number of pregnancies of 13 Dutch IVF clinics from 2004. We calculated the Expected Rank (ER), an estimate of the true rank of a provider, accounting for natural variation. We rescaled the ER to obtain the Percentile based on ER (PCER), that can be interpreted as the probability that a clinic is worse than a randomly selected other clinic. We also calculated a measure for rankability ρ, which is the part of variation between providers that is due to true differences (as opposed to natural variation).

**Results:**

The expected ranks ranged from 1.4 to 11.9 instead of the original ranks 1–13. The ER showed that some clinics performed very similar, which would be disregarded when using standard ranks. The PCER ranged from 7% to 88%. Rankability was substantial (ρ = 0.9)

**Conclusion:**

The Expected Rank provides a way to combine the attractiveness of a ranking, a single number and easy interpretation, with reliable analyses that does justice to the providers, and also allows individual comparisons.

## Background

There is an increasing interest in measuring quality of health care. Data on quality of care often include outcome measures, such as mortality. The idea behind collecting and publishing information on quality of care is that the performance of providers should be measured, as stakeholders such as government, patients, and insurance companies, have a right to know what the services are achieving (an external purpose). Another reason for the continuing demand to broadcast health figures is that publication is thought to act as an incentive for low performers to adopt best practices from the top of the league in pursuit of improvement (an internal purpose). Specifically, rankings may be made according to provider performance. Such ranking was already done in 1995 for physician-specific mortality after coronary-artery bypass grafting surgery in New York State.[1] Currently, ranking health care providers is very popular in the lay press.[2]

In this paper, we use data from IVF clinic rankings based on pregnancy rates. In IVF, the competition to improve results and ranking is even stronger because a large proportion of IVF treatments take place in the private sector.

The pregnancy rate is however influenced not only by quality differences, but also by case mix and natural variation. Natural variation means variation caused by chance, and can also referred to as random variation or unavoidable variation. Case mix and natural variation obscure assessment of the true performance of a clinic and may make standard rankings meaningless. To do justice to the clinics, and to allow patients to make evidence based choices between clinics it is necessary to correct for case mix (such as age of the woman or number of previous cycles of IVF) and to take into account the natural variation that is caused just by chance.[3,4]

We here focus on the natural variation. Different approaches have been suggested. Lemmers et al recently proposed a best-case and worst-case scenario ranking and illustrated this approach on the dataset we also use in this paper.[5] Marshall et al. calculated 95% confidence intervals to the ranks.[6] The aim of this study is to present another method: the Expected Rank (ER), which prevents over interpretation of the rankings. We present our method using IVF data from 13 clinics in the Netherlands.

## Methods

### Data

Data were obtained from a paper of Lemmers at al and came originally from the Dutch Society for Obstetrics and Gynaecology (NOVG). The NVOG monitors clinics in the Netherlands that are licensed to carry out IVF. Every year, records are kept at each clinic of the number of treatment cycles started, the number of pregnancies, singleton ongoing pregnancies, twin ongoing pregnancies and triplet ongoing pregnancies. A pregnancy is defined as a positive test in urine or serum (.50 IU l21), not earlier than 15 days after the ovum pickup. We used the results of treatment cycles that started in 2004.

### Statistical analysis

The differences in pregnancy rates between clinics can be estimated with a fixed or a random effect logistic regression model. For the fixed effect analysis, we fitted a standard logistic regression model, with clinic as a categorical variable. We estimated a coefficient for the pregnancy rate for each clinic (θ_i_) compared to the average using an offset variable. Next, we fitted a random effect logistic regression model. Unlike fixed effect models, random effect model estimators implicitly account for the fact that the observed outcomes for smaller hospitals can take on extreme values because of natural variation rather than an underlying extreme effect. These models estimate the 'true' coefficient for an individual clinic and the 'true' heterogeneity between clinics. With true heterogeneity in this case we mean the variation that is not explained by natural variation, in the literature also referred to as unexplained heterogeneity. [7,8] The true or unexplained heterogeneity is indicated by τ^2^, the variance of the random effects, which can be obtained from the output of random effect models. In the analysis we interpreted classical empirical Bayes parameters and estimates within a Bayesian framework.

### Ranking and rankability

To also account for natural variation in ranking, we calculated the expected rank (ER).[9] The ER is determined by the probability that the performance at a certain clinic i is worse than in another randomly selected clinic. As an intermediate step we calculate the probability that clinic i is worse than clinic k (p(θ_i _< θ_k_|data)), i.e. that the coefficient for the pregnancy rate is smaller in clinic i given the data. We calculate p(θ_i _< θ_k_|data) from the standardized difference in the logistic regression coefficients. The coefficients can be obtained both from the fixed and the random effect models; we use the random effect coefficients.

were Φ is the normal cumulative distribution function. p(θ_i _< θ_k_|data) incorporates both the magnitude  and the uncertainty  of the estimated difference between two clinics. In the random effects approach  and var() are the posterior emperical Bayes estimate and corresponding posterior variance. The expected rank ER is calculated by taking from one particular clinic the comparisons with all the other ones and sum up the probabilities. This sum is than added to 1 to avoid ERs below 1.

We can scale the expected ranks ER between 0 and 100% with percentiles based on expected rank (PCER) for easy interpretation and to make the ranks independent of the number of clinics.

where N is the number of clinics.

The PCER can be interpreted as the probability (as a percentage) that a clinic is worse than a randomly selected other clinic.

To see whether it makes sense to rank the clinics, we can calculate the 'rankability' ρ.

The rankability relates the heterogeneity τ^2 ^from the random effect models (How large are the differences between the clinics?) to the standard error s_i_^2 ^of the individual pregnancy rate coefficients from the fixed effect model (How certain are the differences?). In linear regression ρ would be equal to the intraclass correlation. The rankability can be interpreted as the part of heterogeneity between the clinics that is due to true differences (as opposed to 'natural variation').

The statistical analysis was performed with R statistical software (version 2.5).

## Results

The number of treatment cycles varies from 180 in clinic J to 1264 in clinic A, and the pregnancy rate varied from 32.4 in clinic A to 14.8 in clinic M. We can simply rank the centers based on the actual pregnancy rate, which results in a ranking with clinic A on top of the ranking (rank 1) and clinic M at the bottom (rank 13). The ranking does not change when the data are analyzed with a random effect analysis (Table 1).

**Table 1:** Number of treatment cycles, number of pregnancies, pregnancy rate, rank, expected rank and percentile based on expected rank for each clinic

Clinic	Treatment cycles	Pregnancies	Pregnancy rate	Rank fixed/random	ER	PCER
A	1264	409	32.4	1	1.4	7
B	1027	321	31.3	2	2.0	11
C	1513	453	29.9	3	3.2	20
D	525	154	29.3	4	5.0	35
E	654	186	28.4	5	5.1	35
F	285	81	28.4	6	6.5	46
G	539	177	27.7	7	8.1	59
H	399	102	25.6	8	8.5	61
I	775	182	23.5	9	8.7	63
J	180	41	22.8	10	9.4	68
K	817	164	20.1	11	10.2	75
L	688	119	17.3	12	11.1	82
M	412	61	14.8	13	11.9	88

The Expected Ranks differ from the simple ranks, in that they are shrunken towards the median rank of 7. Clinic A has an ER of 1.4, clinic M has an ER of 11.9. The Percentiles based on Expected Rank, that can have a range from 0% to 100%, vary from 7% for clinic A to 88% for clinic M. This means that clinic A has only a 7% probability to perform worse than another randomly selected clinic. Clinic M has a 88% probability to perform worse than another randomly selected clinic.

**Figure 1:**
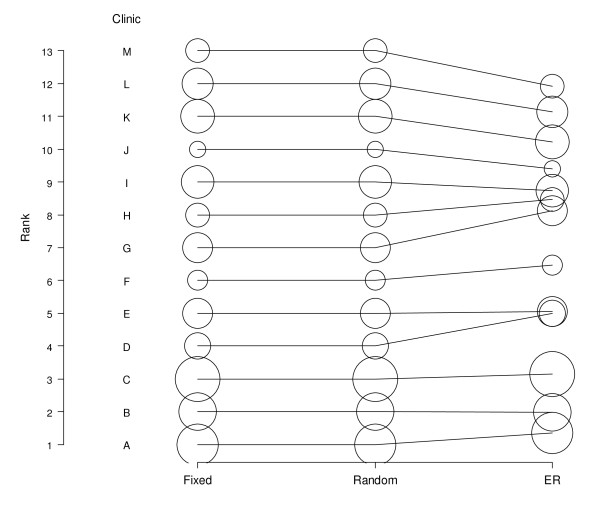
**Rank for each clinic based on fixed effect estimates, random effect estimates and expected rank**.

Figure 1 shows the ranks based on fixed and random analyses and the ER. The dot size indicates the number of treatment cycles. As one could expect, large clinics change less than smaller clinics.

Table 2 shows all the individual comparisons between the clinics. For example, the probability that clinic A performs worse than clinic D is 17%. The probability that clinic G performs worse than clinic F is 54%. The probability that clinic M performs worse than clinic J is 98%.

**Table 2:** Probability that performance in the 'column clinic' is worse than in the 'row clinic'.

Clinics	A	B	C	D	E	F	G	H	I	J	K	L	M
A	-	64%	78%	83%	89%	88%	93%	98%	100%	99%	100%	100%	100%
B	36%	-	65%	73%	81%	81%	86%	95%	99%	98%	100%	100%	100%
C	22%	35%	-	61%	70%	71%	77%	91%	98%	96%	100%	100%	100%
D	17%	27%	39%	-	69%	61%	67%	84%	95%	92%	100%	100%	100%
E	11%	19%	30%	41%	-	54%	58%	79%	93%	90%	100%	100%	100%
F	12%	19%	29%	39%	46%	-	54%	74%	89%	86%	99%	100%	100%
G	7%	14%	23%	33%	42%	46%	-	72%	9%	86%	99%	100%	100%
H	2%	5%	9%	16%	21%	26%	28%	-	73%	71%	95%	99%	100%
I	0%	1%	2%	5%	7%	11%	10%	27%	-	52%	88%	98%	99%
J	1%	2%	4%	8%	10%	14%	14%	29%	48%	-	81%	95%	98%
K	0%	0%	0%	0%	0%	0%	1%	5%	12%	19%	-	83%	93%
L	0%	0%	0%	0%	0%	0%	0%	1%	2%	5%	17%	-	73%
M	0%	0%	0%	0%	0%	0%	0%	0%	1%	2%	7%	27%	-

E.g. there is a 36% probability that an average couple at clinic A has a lower chance of pregnancy than if they attend clinic B.

The heterogeneity between the clinics τ^2^, obtained from the random effect models, was 0.08. The corresponding 95% range of odds ratios was 0.57 to 1.74, meaning that clinics at the higher end of the pregnancy rate distribution have a 1.74 times higher chance of pregnancy than the average. Similar, clinics at the lower end have a 0.57 times smaller chance. The uncertainty measured by the median s_i_^2 ^from the fixed effect model was 0.008. This resulted in a substantial rankability (ρ = 0.9). So 90% of the observed differences between IVF clinics was actually due to 'true' differences.

## Discussion

We made rankings of 13 IVF clinics in the Netherlands, based on pregnancy rates. We calculated the Expected Rank and the Percentiles based on Expected Ranks, to incorporate both the magnitude and the uncertainty of the differences in pregnancy rates between the clinics.

When we want to measure provider performance with outcome measures, in this case pregnancy rates of IVF clinics, two issues are important: case-mix adjustment and natural variation.[3,4] Regarding the first issue, the chance of pregnancy is not only determined by the performance of the clinic but also by characteristics of the mother. When different clinics treat different patients this can already cause variation in pregnancy rate that the clinics can not prevent. Therefore adjustment for case-mix is very important, but was not in the scope of this study. It is however technically readily possible to calculated the case-mix adjusted ER. The only difference is that we obtain the pregnancy rate coefficients from a (random effect) logistic regression model that includes the relevant patient characteristics as explaining variables.

The second issue is natural variation that exists just by chance. Random effect models allow imprecisely estimated outcomes from smaller clinics to 'borrow' information from other clinics, causing their estimates to be shrunk toward the overall mean. Each estimate reflects a compromise between the clinic-specific mean and the overall mean based on the relative magnitude of the variance within a clinic to the total variance (between and within the clinics). Random effect estimates can be considered as the 'true' pregnancy rate coefficients, beyond natural variation.[8] In our study sample size was large. Hence there were no large differences between fixed and random effect analyses, and the ranking did not change.

We used R to calculate the random effect estimates. It is however not always straightforward to derive these and to select the correct information from the output of the various statistical programs. SPSS for example does not fit random effect logistic regression models. Other packages such as SAS and Stata do.

Simple rankings, based both on fixed and random estimates of the pregnancy rate coefficients, disregard both the magnitude and the uncertainty of the differences between clinics. With the expected rank (ER) however, both are incorporated in the ranking. We see that the best clinic has an ER of 1.4 instead of 1, the worst clinic has an ER of 11.9 instead of 13. We also see that some clinics perform in a very similar fashion, such as clinic D and E. The magnitude of the difference is disregarded in the simple ranking (clinic D rank 4, clinic E rank 5), but shown in the ER (clinic D ER 5.0, clinic E ER 5.1). We also see that there is more uncertainty about the performance of the smaller clinics like H and J. This uncertainty is disregarded in the simple rankings (clinic H rank 8, clinic J rank 10) but included in the ER (clinic H ER 8.5, clinic J rank 9.4) So the ER is much more subtle than the simple ranking. For ease of interpretation we calculated the percentile based on expected rank (PCER), which is independent from the number of centers in the sample and indicates the probability that a hospital is worse than a randomly selected other hospital.

Approaches similar to the ER have been proposed by others. Lemmers et al. proposed a best and worst case ranking and Spiegelhalter et al proposed a 95% confidence interval around the ranks.[5,6] Both methods provide 3 numbers: a 'point estimate' and the uncertainty around this estimate. The ER consists of only one number, which is an advantage in our perspective because it is generally easier to process one number than three numbers. On the other hand, the ER does not show the amount of uncertainty, although it is included in the number. The ER also incorporates the magnitude of the difference between the clinics, this is not directly included in the approaches of Lemmers et al. and Spiegelhalter et al.

Another advantage of the ER is the intermediate step of calculation of the probability that a certain clinic performs worse than another one, for all the clinics. These individual comparisons can be very useful for couples when they want to choose an IVF clinic. For example, we saw that the probability that clinic G performs worse than clinic F is 54%. In decision making this probability can be weighed against possible advantages of clinic G, e.g. distance or familiarity with the hospital. The presentation in a table like table 2 however, is only feasible when the number of clinics to compare is small.

The rankability can be used as an indication to see whether it make sense to rank the clinics at all. In this study rankability was 0.9, which is substantial. 90% of the differences between the clinics can be attributed to true differences, and 10% to random variation. It is however a value judgment what can be considered as a high rankability. We would suggest that a rankability above 0.7 is reasonable. The rankability is a function of the heterogeneity τ^2 ^and the uncertainty of the individual center effects s_i_^2^. In this case the heterogeneity was considerable (τ^2 ^= 0.08) and the uncertainty was small because of the large numbers per clinic (si^2 ^= 0.008).

Because of the relatively small uncertainty in this example, the natural variation is limited and there were no large differences between the standard rankings and the Expected Ranks. This is a result of the data, not of the proposed method. Therefore there might have been examples that would have better demonstrated the features of the method. We nevertheless used this dataset since it was used before to present novel ranking methods.

For comparison, in a study on 10 centers in the Netherlands treating stroke patients, the unadjusted τ2 was 0.38, but rankibility was only 0.55, due to small numbers. The ERs ranged from 1.7 to 8.6 instead of the original ranks 1–10 and six of the ten centers had en ER close to the median rank of 5.5. (Lingsma et al, How to compare center based on outcome: results from the Netherlands Stroke Survey, submitted).

Regarding the communication of performance measures, rankings may be not the ideal way to present the data in all its subtleties, they are however attractive to the press and the public. Our proposed measure, the Expected Rank, provides a way to combine the attractiveness of a ranking, a single number and easy interpretation with reliable analyses that does justice to the providers, and also allows individual comparisons.

## Conclusion

In comparing health care providers, the Expected Rank combines the attractiveness of a ranking, a single number and easy interpretation, with reliable analyses that does justice to the providers, and also allows individual comparisons.

## Competing interests

The authors declare that they have no competing interests.

## Authors' contributions

HL did the statistical analyses and drafted the manuscript. RE participated in the statistical analyses. ES conceived of the study, and helped to draft the manuscript. All authors read and approved the final manuscript.

## Pre-publication history

The pre-publication history for this paper can be accessed here:

http://www.biomedcentral.com/1471-2288/9/53/prepub
